# New perspectives in application of kidney biomarkers in mycotoxin induced nephrotoxicity, with a particular focus on domestic pigs

**DOI:** 10.3389/fmicb.2023.1085818

**Published:** 2023-04-14

**Authors:** Zsolt Ráduly, András Szabó, Miklós Mézes, Ildikó Balatoni, Robert G. Price, Mark E. Dockrell, István Pócsi, László Csernoch

**Affiliations:** ^1^Department of Physiology, Faculty of Medicine, University of Debrecen, Debrecen, Hungary; ^2^ELKH-DE Cell Physiology Research Group, University of Debrecen, Debrecen, Hungary; ^3^Doctoral School of Molecular Medicine, University of Debrecen, Debrecen, Hungary; ^4^Agrobiotechnology and Precision Breeding for Food Security National Laboratory, Department of Physiology and Animal Health, Institute of Physiology and Nutrition, Hungarian University of Agriculture and Life Sciences, Kaposvár, Hungary; ^5^ELKH-MATE Mycotoxins in the Food Chain Research Group, Kaposvár, Hungary; ^6^Department of Food Safety, Institute of Physiology and Nutrition, Hungarian University of Agriculture and Life Sciences, Gödöllő, Hungary; ^7^Clinical Center, University of Debrecen, Debrecen, Hungary; ^8^Department of Nutrition, Franklin-Wilkins Building, King’s College London, London, United Kingdom; ^9^SWT Institute of Renal Research, London, United Kingdom; ^10^Department of Molecular and Clinical Sciences, St. George’s University, London, United Kingdom; ^11^Department of Molecular Biotechnology and Microbiology, Institute of Biotechnology, Faculty of Science and Technology, University of Debrecen, Debrecen, Hungary

**Keywords:** nephrotoxicity, KIM-1, kidney biomarkers, mycotoxins, animal toxicity, NAG, NGAL biomarkers, mycotoxin induced nephrotoxicity

## Abstract

The gradual spread of *Aspergilli* worldwide is adding to the global shortage of food and is affecting its safe consumption. *Aspergillus*-derived mycotoxins, including aflatoxins and ochratoxin A, and fumonisins (members of the fusariotoxin group) can cause pathological damage to vital organs, including the kidney or liver. Although the kidney functions as the major excretory system in mammals, monitoring and screening for mycotoxin induced nephrotoxicity is only now a developmental area in the field of livestock feed toxicology. Currently the assessment of individual exposure to mycotoxins in man and animals is usually based on the analysis of toxin and/or metabolite contamination in the blood or urine. However, this requires selective and sensitive analytical methods (e.g., HPLC-MS/MS), which are time consuming and expensive. The toxicokinetic of mycotoxin metabolites is becoming better understood. Several kidney biomarkers are used successfully in drug development, however cost-efficient, and reliable kidney biomarkers are urgently needed for monitoring farm animals for early signs of kidney disease. β_2_-microglobulin (β_2_-MG) and *N*-acetyl-β-D-glucosaminidase (NAG) are the dominant biomarkers employed routinely in environmental toxicology research, while kidney injury molecule 1 (KIM-1) and neutrophil gelatinase-associated lipocalin (NGAL) are also emerging as effective markers to identify mycotoxin induced nephropathy. Pigs are exposed to mycotoxins due to their cereal-based diet and are particularly susceptible to *Aspergillus* mycotoxins. In addition to commonly used diagnostic markers for nephrotoxicity including plasma creatinine, NAG, KIM-1 and NGAL can be used in pigs. In this review, the currently available techniques are summarized, which are used for screening mycotoxin induced nephrotoxicity in farm animals. Possible approaches are considered, which could be used to detect mycotoxin induced nephropathy.

## 1. Introduction

Mycotoxins are low-molecular-weight naturally occurring organic contaminants and are secondary metabolites of filamentous fungi, mainly *Aspergillus*, *Penicillium,* and *Fusarium* (Bennett and Klich, 2003). Their presence cause damage to agricultural products and they can persist in the food supply chain (Ganesan et al., 2022). Increased temperature, elevation in carbon dioxide, and extremes in water availability can influence the occurrence and/or frequency of mycotoxin production. This accumulates in food mainly cereal grains, which increases the risk of dietary contamination (Perrone et al., 2014). *Aspergillus*, *Penicillium*, *and Fusarium* spp. usually infect cereal grains (Udomkun et al., 2017). They can occur in the field before and after harvest as well (Patriarca and Fernández, 2017). Alternative and effective pre- and post-harvest strategies should be used to minimize contamination in food and feed products to ensure that mycotoxin levels are below the regulated limits for safety (Prietto et al., 2015). Modern management methods for *Aspergillus* mycotoxin are oriented toward investigating the fungal diversity and the population distribution of *Aspergillus* spp. (Battilani et al., 2006). Mycotoxins can enter the food chain in the field during storage or later. The production of mycotoxins usually occurs at the pre-harvest stage and their accumulation increases after harvest. The best way to prevent mold infection and mycotoxin production is by keeping optimal storage conditions for agricultural products (Torres et al., 2014). Moreover, Good Agricultural Practices (GAPs) should be followed wherever agricultural production is carried out (Sakuda et al., 2014). *Aspergillus* is an airborne and soil-borne fungus; strategies should minimize the survival of *Aspergillus* in weeds close to the crop, which reduces the possibility of mycotoxin contamination (Abbas et al., 2009). During cultivation, fertilizers should be applied, and soil chemical properties, such as pH and organic carbon content of soil, should be monitored. Adequate plant protection and harvest time are important factors in cereal cultivation and provides a simple way to reduce mycotoxin contamination (Leslie et al., 2021; Keszthelyi et al., 2022). Additionally, different cultivars are defined by yield, sensitivity to abiotic factors like temperature or water deficiency, and sensitivity to biotic factors like plant pathogens and pests, which result from mycotoxin contamination (Afolabi et al., 2007). Contamination with *Aspergillus* mycotoxins of cereal grains in Europe in the first quarter of 2022 was moderate (DSM, 2022). The prevalence of aflatoxins (AFs) was 32%, and ochratoxin A (OTA) 11% in positive samples. The average contamination level of AFs was 127 μg/kg, and OTA was 9 μg/kg. Regulated levels in cereal grains used for animal feeds for AFs is 20 μg/kg [Regulation EC 574/2011 (EU—COM 2014/015, 2014)] and 250 μg/kg for OTA [Recommendation EC 2006/576; EC (European Comission), 2006]. Besides the appropriate pre- and postharvest technologies toxic effects of mycotoxins can be mitigated with different mycotoxin binding agents (Kolawole et al., 2022), such as algal polysaccharides (Guo et al., 2021; Liu et al., 2022b) or biodegradation by probiotic bacteria (Park et al., 2022). Additionally, antioxidants can reduce some toxic effects of mycotoxins (Liu et al., 2022a,b). Unlike *Aspergillus*, which is more widespread in warmer climates *Penicillium* is more significant in regions with a temperate climate and is endemic in Northern Europe and Canada (Awuchi et al., 2021). In addition to OTA, *Penicillium* can produce Citrinin (CIT). *Penicillium*-produced CIT and OTA occur most commonly during storage (Kamle et al., 2022). In contrast, *Fusarium* mycotoxin generation, occurs predominantly in the field (Birr et al., 2021). *Fusarium verticillioides* is generally associated with maize but it is also capable of asymptomatic infections in other monocots and even sugar beet (Blacutt et al., 2018).

AFs caused the first well documented mycotoxin induced toxicosis in a turkey farm in England, where 100,000 turkeys died in 1960 (Nesbitt et al., 1962; Pickova et al., 2021). From the 1970’s, there are reports that the mycotoxin induced nephropathy was present in 40–60/100,000 pigs in Denmark, similar reports have been published from all over Europe. Furthermore, among farm animals, pigs are the most susceptible to the accumulation of OTA and consequent kidney damage (Kępińska-Pacelik and Biel, 2021). The objective of this review is to summarize the current literature on mycotoxin induced nephropathy, particularly in domesticated pigs, focusing on kidney biomarkers monitored by different analytical methods.

## 2. Kidney and mycotoxins

### 2.1. Mycotoxin exposure and general introduction of nephrotoxic mycotoxins

**Figure 1:**
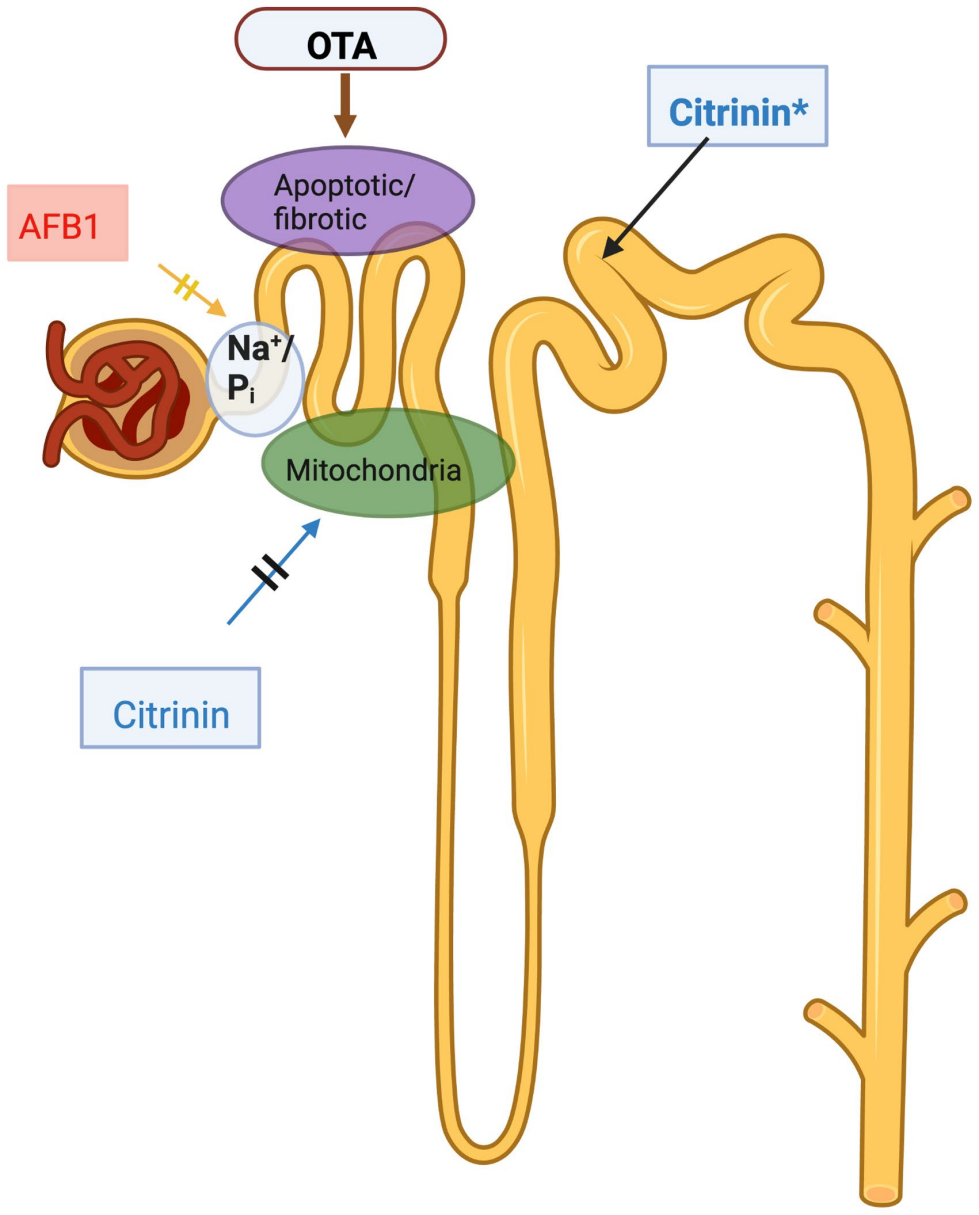
Mycotoxins affecting kidney, a schematic illustration. AFB1 dose dependently reduces Na^+^/P_i_ co-transport in proximal tubule epithelial cells, reducing reabsorption of inorganic phosphates; OTA also targets the proximal tubule, causing cell death and profibrotic effects; citrinin targets the proximal tubule in many species, such as dogs, pigs, rabbits and rats but in mice citrinin targets the distal tubule (indicated by asterisk in the figure). The figure was created with BioRender.com.

AFs are considered genotoxic mycotoxins and no exposure level is considered safe. The more frequent subtypes are AFB1, AFB2, AFG1, AFG2. A toxic metabolite of AFB1, AFM1 is excreted through milk and its presence in milk and milk products, causes a significant food safety problem. AFB1 is the most toxic of known aflatoxins (Manafi et al., 2011; Rushing and Selim, 2019). AFs modify the expression of various genes associated with fatty acid metabolism and energy production at the cellular level (Kumar et al., 2017). They also cause downregulation of antioxidant defense (Yarru et al., 2009). Hepatotoxic effects in humans and experimental animals have been described. AFs are well-known hepatocarcinogens in laboratory animals, and there is epidemiological evidence linking human exposure and hepatocellular carcinoma; it is classified as Group 1 human carcinogen (International Agency for Research on Cancer, 2012). The potential mechanisms of AFB1 and AFM1 subacute toxicity in the kidney were studied in a mouse model. Results revealed that AFB1 or AFM1 activated oxidative stress and caused renal damage, particularly in the proximal tubule (see Figure 1). Proline dehydrogenase (PRODH) and pro-apoptotic factors (Bax, Caspase-3) were upregulated, while the apoptosis inhibitor Bcl-2 was downregulated in mRNA and protein expression (Li et al., 2018). Ochratoxins are a group of mycotoxins produced by *Aspergillus* species, e.g., *Aspergillus niger* as well as some *Penicillium* which can exert damage to organisms (Ringot et al., 2006). For example, OTA disrupts several cell functions, including cell proliferation, division, and signaling pathways (Vettorazzi et al., 2013; Hassan et al., 2022), it also has a synergistic effect on other co-occurring mycotoxins (Liu et al., 2022a).

Dietary exposure to OTA causes severe health problems in animals and humans, including poultry ocochratoxicosis, porcine nephropathy, human endemic nephropathies, and urinary tract tumors (Heussner et al., 2015). As OTA has showed carcinogenic effects, it has been classified as Group 2B human carcinogen (Ostry et al., 2017). Besides its carcinogenic and nephrotoxic effects, hepatotoxic, teratogenic, neurotoxic, genotoxic, and immunotoxic effects have also been described in animals (Marin-Kuan et al., 2006; Solcan et al., 2013; Vlachou et al., 2022). CIT often found together with OTA and can be analyzed together. According to the classification of IARC, CIT is a group 3 human carcinogen which means that CIT is not classified in terms of its carcinogenicity in human (Ali and Degen, 2019). However, CIT of special interest in pigs and poultry, as their diet are normally based on cereals and other grains. It is produced by several species in the genera *Monascus, Aspergillus* and *Penicillium*, and it occurs principally in plant products and stored grains. CIT has a “level of no concern for nephrotoxicity”—a provisional tolerable daily intake (PTDI) value of 0.2 μg/kg bw (Leszkowicz et al., 2008; Flajs and Peraica, 2009). CIT and OTA have also been associated with alterations in renal function and/or with the development of renal pathologies (Ostry et al., 2013). The exact mechanism of CIT induced nephropathy is not fully understood and is species specific (see Figure 1), however, several hypotheses exist. CIT could target calcium homeostasis of the kidney cells. Several animal studies have been carried out, e. g. CIT treatment resulted in swollen and degenerate mitochondria in renal cortical cells of broiler chickens, pigs and laying hens as well (Meerpoel et al., 2020). CIT tends to accumulate in different tissues of pigs and poultry; however, a 3 week-long treatment caused no toxicological problem (Meerpoel et al., 2020).

Fumonisin mycotoxins are mainly produced by filamentous fungi *Fusarium verticillioides* and *Fusarium proliferatum* and provide at least 28 conformational analogues; these are classified into the sub-groups, A, B, C, and P series (Rheeder et al., 2002). From a toxicological aspect the B series, in particular fumonisin B_1_ (FB_1_) is the most challenging, exerting species-specific and target organ specific effects (Chen et al., 2021; Guerre et al., 2022).

Due to the variety of feedstuffs used in the pig industry, multiple fungi contamination has been detected, so several different types of mycotoxin toxicity occur (Holanda and Kim, 2021). It is known that the toxic effects of several different mycotoxins together are stronger because it has been shown that although the individual level of toxins are below the governmental guidelines the total effect can be above (Holanda and Kim, 2021).

## 3. Animal and cellular models of nephrotoxicity

The kidney is a multifunctional organ, containing the nephrons which are its structural and functional units (Wasung et al., 2015). A variety of different cells in the nephron perform highly complex and precisely organized biological processes. Any trauma on these cells can induce kidney damage and associated cardiovascular disease as well as metabolic disorders, resulting in subsequent endocrine dysfunction, and further decline toward renal failure. Owing to the high blood flow in the kidney and its unique metabolism, it is particularly susceptible to nephrotoxic attack (Nigam et al., 2015). Environmental pollutants which target the kidney include metals, solvents as well as naturally occurring compounds, including aristolochic acid and mycotoxins (Vanmassenhove et al., 2013; Weidemann et al., 2016).

### 3.1. Nephrotoxicity in animals

**Figure 2:**
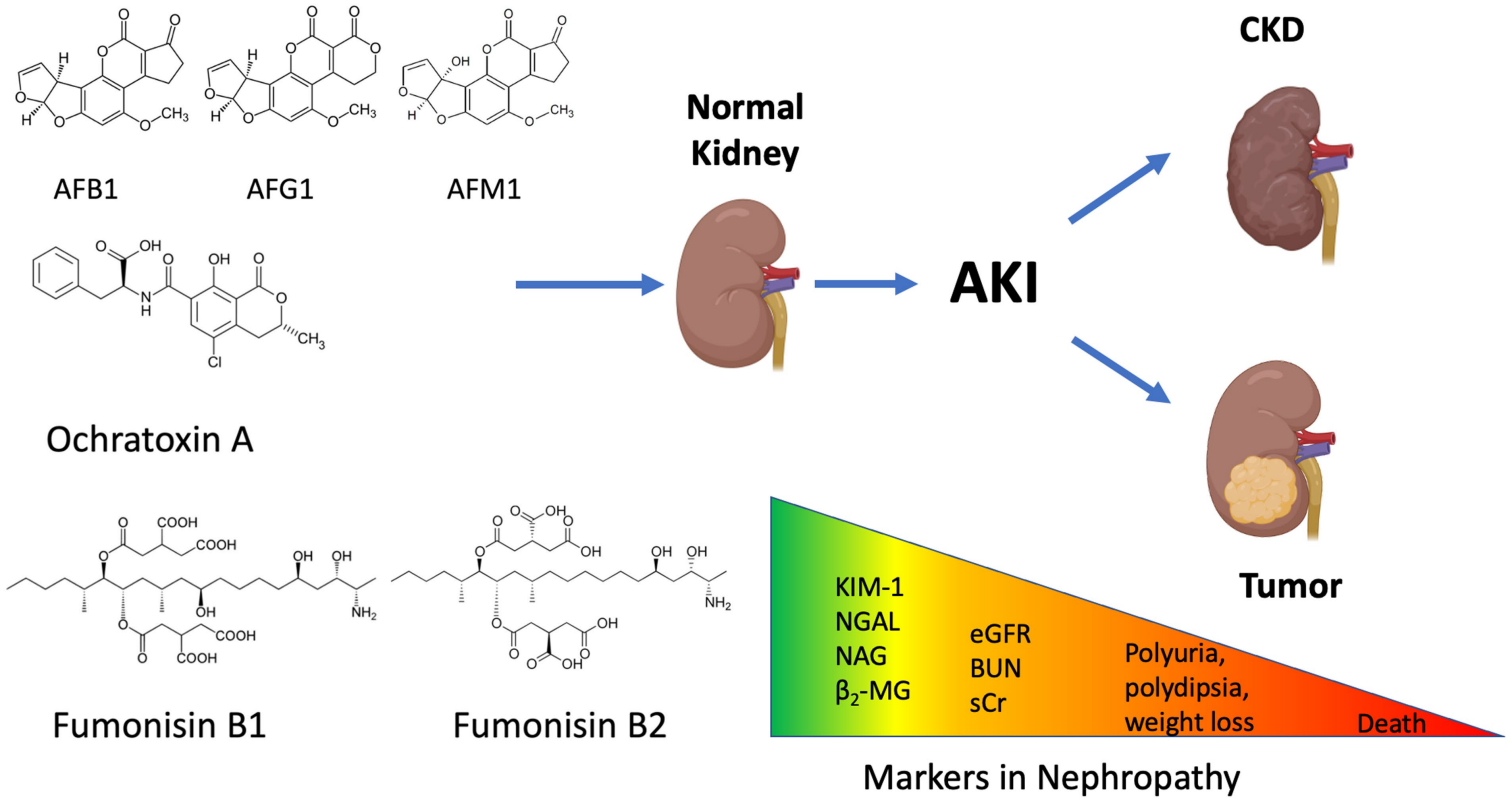
Mycotoxin induced nephropathy and the possibilities for intervention. The different types of mycotoxins could cause the deterioration of kidney function. In order to prevent acute kidney injury (AKI), novel kidney biomarkers should be applied to screen the affected animals. Detecting the traditional markers (eGFR: estimated Glomerular Filtration Rate; BUN: Blood Urea Nitrogen; sCr: serum Creatinine) are often too late to act and treat the animals. The figure was created with BioRender.com.

Acute kidney injury (AKI) is a frequently occurring kidney problem in humans, and farm animals as well (Schiffl and Lang, 2012). Early identification, precise veterinary diagnosis and appropriate treatment are therefore needed. Renal problems could cause significant weight loss, lethargy and decreased meat production, e.g., in pigs or poultry (Vlachou et al., 2022). Estimated glomerular filtration rate (eGFR), blood urea nitrogen (BUN) and serum creatinine (sCr) have been used to detect kidney failure as biomarkers in routine laboratories, however, these are unspecific and occur late in the development of the disease (see Figure 2; Wasung et al., 2015; Hall et al., 2021). Early detection of AKI, is preferred and as a consequence more sensitive diagnostic markers are needed. There is little data in the literature, where early markers have been used to detect mycotoxin induced nephropathy (Elling et al., 1985; Hoffmann et al., 2010a; Musiał, 2021). The most visible sign of nephropathy in animals is retarded growth, and at this timepoint is often too late to treat the animals. Traditional methods to used estimate kidney function, e.g., eGFR, sCr and BUN need to be supported by more sensitive markers such as the more recently described biochemical biomarkers (see Figure 2; Wasung et al., 2015; Malir et al., 2019).

### 3.2. Novel nephrotoxicity biomarkers analyzed in animals

Various animal models are widely used to test the nephrotoxic effects of many pharmacologically active substances. Renal biomarkers are defined in terms of their chemical, structural, physiological characteristics. That show the progress, severity or presence of any damage within the kidney that alters its function. As an example, urinary biomarkers including NAG are suitable to estimate the incidence and size of drug-induced kidney injuries (Hoffmann et al., 2010a; Sheira et al., 2015). According to Vaidya et al. (2010) urinary KIM-1 excretion outperforms other markers in rats exposed to different agents including Vancomycin, Tacrolimus, Gentamicin, Cisplatin (proximal tubular toxicants); Puromycin, Doxorubicin (glomerular toxicants); Lithium, Furosemide (tubular and collecting duct toxicants); α-Naphthyl isothiocyanate (ANIT) and Methapyrilene (hepatotoxicants; Vaidya et al., 2010). Moreover, urinary NAG is one of the most frequently used biomarkers to detect renal tubulopathy in both human and animal studies (Sheira et al., 2015), however the correlation needs to be precisely defined.

In rat models, puromycin aminonucleoside (PAN) or cisplatin (CDDP) induce glomerular and proximal tubular injuries, respectively. PAN increased urinary NGAL and albumin levels (dysfunction of proximal tubule) meanwhile CDDP was detected with highest sensitivity by KIM-1 (Tonomura et al., 2010). Lactate dehydrogenase (LDH) was applicable for the detection of a wide spectrum nephron injuries (Tonomura et al., 2010). In a large-scale comparative study, the following biomarkers were tested and compared: total protein, albumin, KIM-1 clusterin, β_2_-MG, Cystatin-C, alpha-glutathione S-transferase, mu-glutathione S-transferase, NAG, aspartate aminotransferase and NGAL (Tonomura et al., 2010).

Urinary cystatin-C levels are indicative of early acute kidney injuries provoked by, e.g., cisplatin treatments in rats although the change ratios for urinary KIM-1, GST-α, and EGF were higher. Furthermore, other biomarkers, including β_2_-MG, calbindin, clusterin, GST-μ, NGAL, osteopontin, TIMP-1, and VEGF have been also tested (Togashi et al., 2012).

Animal models other than rats are also used to estimate nephrotoxicity triggered by aminoglycosides including pigs (e.g., tobramycin; Lipcsey et al., 2009), cynomolgus monkeys (gentamicin; Gautier et al., 2016); dogs and mice (gentamicin; Al Suleimani et al., 2018). In a most recent study, 10 urinary biomarkers (albumin, clusterin, cystatin C, KIM-1, NGAL, liver-type fatty acid-binding protein, NAG, osteopontin, retinol binding protein 4 and total protein) were assayed to detect nephrotoxicity elicited by 5 nephrotoxicants (cefpirome, cisplatin, naproxen, cyclosporine, and a combination of gentamicin with everninomicin) in primates. KIM-1 and clusterin showed the best overall performance across these studies (Vlasakova et al., 2020). There were differences in the histopathological patterns, which could be related to tubular injury severity and recovery potential, underlying histopathologic processes, biomarker response and the resulting prognosis (Vlasakova et al., 2020). The biomarkers discussed above are biomarkers of effect, that is they are markers of damage caused by nephrotoxins. As can be seen in Figure 1, different mycotoxins target different regions of the nephron and hence, as with other nephrotoxins, one can differentiate between damage to distinct regions of the tubule. As other nephrotoxins may also cause similar damage, the expression of the biomarkers themselves does not conclusively prove exposure to particular mycotoxins, they indicate renal damage. Combined measurement of biomarkers of effect and biomarkers of exposure would provide a more precise diagnosis. A number of recent studies in humans have measured urinary aflatoxin to determine exposure in at risk populations (Ezekiel et al., 2018; Ferri et al., 2020; Nasir et al., 2021). Although, this would not prove that the mycotoxin caused the renal damage, a correlation between a biomarker of effect and a biomarker of exposure would provide good evidence.

## 4. Biomarker approaches, which could be used for the detection of mycotoxin induced AKI and CKD in animals

### 4.1. Molecular mechanisms underlying mycotoxin induced nephrotoxicity

The effects of AFs and OTA depend on their accumulation in particular organs or tissues. Accumulation of OTA in the kidney was higher than AFs, possibly due to their rapid metabolism (Corcuera et al., 2015), and its accumulation correlates with kidney injury. Renal function in chronic aflatoxicosis was determined in laying hens. AFs exposure increases Ca^2+^, Na^+^, phosphate fraction excretion, and glomerular filtration rate also reduces (Martínez-de-Anda et al., 2010). OTA-induced toxicity was investigated in an *in vitro* model using human proximal tubule HK-2 cells. OTA decreased cell viability but increased the expression of KIM-1 (Schulz et al., 2018).

OTA treatment increased intracellular reactive oxygen species (ROS) and malondialdehyde and decreased glutathione levels. The gene expression of aryl hydrocarbon receptor (AhR) and pregnane X receptor (PXR) as well as some cytochrome genes (CYP1A1, CYP1A2, and CYP3A4) of the phase I xenobiotic transformation were induced by OTA exposure. Moreover, the mRNA expression of phase II enzymes such as heme oxygenase-1, NAD(P)H quinone dehydrogenase 1 (NQO1), and glutamate cysteine ligase were upregulated. The response of OTA-orally administered mice also showed marked increases in these enzymes and KIM-1. These results indicate that OTA induces phase I and II enzymes through the AhR, PXR, and Nrf2 signaling pathways in HK-2 cells, which may lead to the modulation of proximal tubule injury (Lee et al., 2018). OTA exposure in mice significantly increased the DNA-damage-inducible transcript 3 (gadd 45) result in growth arrest and DNA-damage-inducible 45 alpha (gadd 153) mRNA levels. High dosage of acute OTA administration elevated the mRNA expression of annexin2 and clusterin but decreased the sulfotransferase 1C2 (sult1c2) mRNA level (Ferenczi et al., 2014). Repeated oral dose of OTA induced oxidative stress in mice kidneys, which was concluded from increased lipid peroxidation parameters (conjugated dienes and trienes, and malondialdehyde), downregulation of glutathione S-transferase and, upregulation of glutathione peroxidase genes. Expression of the transcription factors of the antioxidant genes, Keap1 and Nrf2, respond differentially to OTA exposure. The gene expression of Keap1 decreased, and the gene expression of Nrf2 increased. However, protein expression of NRF2 and its Ser40 phosphorylated form did not alter (Ferenczi et al., 2020). In a study with porcine kidney cells, AFB1 and deoxynivalenol showed synergistic cytotoxic effect, which suggests the importance of multiple mycotoxicosis even in farm animals as well (Zachariasova et al., 2014). Pigs are the most sensitive farm animal to OTA (Tkaczyk et al., 2021). Progressive mycotoxin-induced porcine nephropathy is seen in pigs with a dietary concentration of 1 mg/kg of OTA. Low levels of OTA could induce polydipsia or polyuria, while higher levels could even provoke vomiting, diarrhea and even death (Tkaczyk et al., 2021). OTA has a high affinity for serum albumin and other macromolecules in the blood, so its half-life is estimated to be more than 72 h. Consequently, it is hard to detect a high concentration of OTA in the urine, even if the blood levels are high as well (Kőszegi and Poór, 2016).

Fumonisins are water soluble and polar, enabling partial urinary excretion (Yang et al., 2022) in monkeys (Shephard et al., 1994), meanwhile providing dose dependent urinary excretion in rats (Cai et al., 2007). From a nephrotoxic viewpoint, it’s not the relatively low urinary excretion rate is critical (Norred et al., 1993), but the biochemical route/mode of action of FB_1_. This leads to marked cellular disturbances, which are dependent on its molecular structure. As FBs are conformational analogs of free sphinganine, competitively inhibiting the ceramide-synthase (CerS) enzymes (Loiseau et al., 2015; Wang et al., 2015), they demonstrate competitive-like kinetic behavior with both sphinganine and stearoyl-CoA, in cell culture (Merrill et al., 1993). As a consequence, the substrates sphinganine (Sa) and, less markedly sphingosine (So) accumulate (Riley and Merrill, 2019) in the affected tissue(s); this accretion is so characteristic for FB_1_ that Sa and So (and their ratio, e.g., in the urine) are accepted as interspecific, sensitive FB_1_ biomarkers, e.g., for humans (Schertz et al., 2018; Wangia et al., 2019); rat (Schertz et al., 2018); horse and pig (Riley et al., 1994). Though FB_1_ exerts organ-specific toxic effects, and mammals and birds are react differently, the Sa/So ratio correlates with liver and kidney toxicity as well it often precedes signs of acute toxicity (Tran et al., 2006). This occurs in in fowl where the kidney is not the primary target organ. However, urinary Sa/So ratio is still an important biomarker, e.g., in ducks (Tran et al., 2006) and in turkeys (Guerre et al., 2022).

BIOMIN reported 64% contamination rate of FB_1_ in grain in 2020 (BIOMIN, 2021) demonstrating how worldwide the problem is. The FB_1_ spoilage/contamination of farm and pet animals’ grain based feed sources (primarily corn), leads to a wide spectrum of symptoms based on disturbances in multiple organs: liver and kidney toxicity is the main characteristic in many species (Riley and Merrill, 2019). Other symptoms are also reported, including the development of tumors in rodents [increased tumor necrosis factor (TNF)-α secretion; Dugyala et al., 1998], vascular and brain dysfunction and equine leukoencephalomalacia (Riley and Merrill, 2019), and porcine pulmonary edema syndrome (Haschek et al., 2001). Meanwhile carnivores are less severely exposed to mycotoxins of grain, complete feed ratios pose risk even in these species (dogs: Ekici and Yipel, 2022; cats: Grandi et al., 2019).

In general, ruminants are treated as less susceptible to fumonisins, since the ruminal microbiota provide a natural barrier with a substantial degradation capacity (Seeling et al., 2005). In fish, because of the sampling uncertainties, urinary biomarkers are not yet been published, but FB_1_ has been tested as a significantly harmful agent (Meredith et al., 1998), and has been found to make intensive perturbations in the plasma concentration and relative ratio of free sphingoid bases (incl. Sa and So).

### 4.2. Kidney biomarkers in detecting mycotoxin induced nephrotoxicity

Until recently the diagnosis of mycotoxin toxicity was mainly based on quantitative determination of mycotoxins in feeds and impairment of the production traits (Heyndrickx et al., 2015; Watson et al., 2018; Ali and Degen, 2020). However, the organ or mycotoxin-specific markers are rare in diagnostic processes except detection of mycotoxins or their metabolites in blood or urine. Noninvasive and specific markers, such as urinary biomarkers, would be effective complementary tools for correctly diagnosing kidney damage (Vanmassenhove et al., 2013; Wasung et al., 2015). Still, there is some limitation to their use due to methodological problems with urine sample collection. It would be useful even in farm conditions for mammals, such as pigs, ruminants, and horses, but it cannot be used in avian species due to cloaca. In pigs, the noninvasive method for urine collection force the pigs to stand up by clapping or shouting. A few minutes later, animals spontaneously show micturition and urine can be collected. In cattle, a noninvasive plastic urine collection device is proposed for females (Lascano et al., 2010), which can replace catheters but allow them to collect urine from the urethra. In horses, most animals urinate soon after standing or shortly after putting them in a freshly bedded stall, and the urine can be collected manually during urination (Schumacher and Moll, 2011). Another methodical problem is the lack of availability of species-specific urinary biomarker as diagnostic kits, except pig-specific KIM-1, however, this problem could be easily solved by molecular biological approaches.

In free-range animals it may be easier to use serum rather than urine to test for biomarkers. A strong correlation between AFB1 intake and serum levels has been demonstrated (Turner and Snyder, 2021). However, in a study of children in Tanzania, only a modest but statistically significant correlation was observed with the AF-alb biomarker and maize-based AF intake (Turner and Snyder, 2021). The same publication reported that “The toxicokinetics of OTA are complex; while rapidly absorbed, OTA is non-covalently associated with serum albumin, with a suggested <0.2% OTA free fraction in serum” indicating that the measure of serum OTA is complex and may be species specific (Turner and Snyder, 2021). Serum levels may be used as markers of exposure but give little data on organ damage. Serum levels of biomarkers of kidney damage are relatively insensitive. Urinary KIM-1 and NGAL have been shown to be markers of kidney damage, however in serum both may indicate non-kidney damage. KIM-1 was first known as TIM-1, T-cell immunoglobulin and mucin-containing molecule. Neutrophil gelatinase-associated lipocalin (NGAL), is an inflammatory marker associated with the pathophysiology of heart failure (HF), the psychopathology of depression and the co-existing symptoms of depression in HF patients (Naudé et al., 2015). Consequently, neither offer much specificity regarding kidney damage.

AFs and OTA toxicity are global problems in agriculture because they contaminate cereal grains which are basic components of animal feed selective biomarkers would therefore provide important tools for diagnosing kidney damage caused by mycotoxins. Urinary parameters, such as polyuria, glucosuria, proteinuria, and osmolality, are generally used as markers of kidney damage in farm animal practice (Elling et al., 1975) but lack specificity. A good correlation has been observed between the amount of mycotoxins ingested and the amount of relevant biomarkers, excreted in urine of pigs (Gambacorta et al., 2013). The nontoxic metabolite of OTA, ochratoxin α, was also detected in pig urine (Tkaczyk et al., 2021). However, the excretion of mycotoxin metabolites is not specific to the kidney and requires specific analytical tools for analyzing, including LC-MS/MS. As the availability of these techniques requires specific laboratories, routine veterinary practice is not available in rural areas. Even in recently published research, a group analyzed the effects of OTA and FB_1_ on mice, however just BUN and sCr were applied to monitor kidney damage (Li et al., 2023). Other, more specific urinary biomarkers, such as KIM-1 and NGAL, were proposed by Chade et al. (2018). These markers are used to detect kidney disease, and have the potential to somehow monitor mycotoxin exposure to, e.g., AFs or OTA. KIM-1 is a sensitive noninvasive urinary biomarker because its gene expression is low in normal kidneys but increases dramatically after proximal tubular cell damage and repair. It can be determined in urine (Ichimura et al., 2004; Hoffmann et al., 2010a). Several other biomarkers were proposed for detecting kidney damage caused by OTA, such as lipocalin-2, tissue inhibitor of metalloproteinases-1 (Timp-1), clusterin, osteopontin, and vimentin, although KIM-1 was the most promising one. However, in *in vitro* studies, these markers were not so suitable for the detection the nephrotoxic effects of OTA (Rached et al., 2008). On the contrary, OTA treatment was applied to mice where KIM-1 and NGAL were used to monitor the nephrotoxicity of the xenobiotics (Hassan et al., 2022). Studies have also been carried out using serum or blood samples collected to determine biochemical parameters which reflect kidney function (Mohd Redzwan et al., 2014). Recently, a pilot study found a good correlation among AFB1 intake and kidney function using urinary KIM-1, and Cystatin-C, in human samples (Díaz de León-Martínez et al., 2019). These markers are sensitive and specific for renal damage, but not specific for the mycotoxin exposure alone. Combining these markers with secreted metabolites in urine or blood could increase their specificity toward mycotoxins.

The most accepted and used urinary biomarkers for FB_1_ among a wide variety of vertebrate species is the free sphinganine and sphingosine ratio, more recently further possible candidate molecules have been suggested. Using LC-TOF (time of flight approach; ceramide-C42 compounds) were found to be of diagnostic value in fibrotic kidneys of mice and humans (Eckes et al., 2021), as well as FB_1_ fed piglets’ liver (Loiseau et al., 2015). However they have not yet been tested as a direct FB_1_ urinary biomarker. Similarly, Loiseau et al. (2015) reported that sphingomyelins (SPM), specifically SPM-d18:1/16:0, SPM-d18:0/18:0, SPM-d18:1/18:0, and SPM-d18:1/24:1 decreased significantly in porcine liver with FB_1_ feeding, while Marczak et al. (2021) found SPM-C39:1 to be indicative of CKD (chronic kidney disease). This requires further testing, to determine whether SPM and possibly ceramide molecules will become alternative biomarkers, besides the most widely accepted Sa/So ratio in pet and farm animals, like in pigs (Marczak et al., 2021). The most sensitive fumonisin toxicity biomarker in pigs (and most mammals) is the free sphinganine/sphingosine ratio in the serum as well (Kim et al., 2006; Burel et al., 2013).

It is important to note that sphingosine and sphinganine analysis needs liquid chromatographic techniques (LC/MS, LC/MS-MS), and further possible candidate compounds need much more sensitive detection (LC-TOF or LC-qTOF). These tests are unsuitable for point of care testing but would be of value as confirmatory laboratory-based tests. So far, just limited data is available in connection with FB_1_ and the emerging kidney biomarkers (NAG, KIM-1, NGAL), but a dose–response experiment on HK-2 kidney cells showed an increased expression of Kim-1 correlated with the mRNA level after FB_1_ treatment (Hou et al., 2021). Anyway, other animal studies are still needed to be carried out to analyze the exact correlation among these biomarkers and FB_1_.

### 4.3. Potential assay procedures for testing mycotoxin induced nephrotoxicity

As mentioned above, the most widely used biomarkers in other fields, are KIM-1, NAG, NGAL, Cystatin-C and β2-MG (Table 1). There is an increasing demand for KIM-1 assays, as this has been shown to be a suitable marker for nephrotoxicity testing of drugs (Wasung et al., 2015; Song et al., 2019). Enzyme-linked immunosorbent assay (ELISA) or microparticle Luminex xMAP Technology assay could be used to measure KIM-1 levels (Vaidya et al., 2009). Different types of assays are available for use in animals, however, some of these procedures have not been modified for the specific determination of mycotoxins. As an example, Pig HAVCR1/KIM-1 Sandwich Elisa KIT is available for the detection of porcine KIM-1 in plasma and serum and other biological fluids (e.g., FineTest ® EP0102 or LSBio Biotechnology ® LS-F36323). A good KIM-1 assay should be specific for the cleaved extracellular domain of the protein. In a mentioned Sandwich Elisa kit, the detection range is between 0.15–10 ng/mL, and the sensitivity is 0.094 ng/mL, in serum or blood samples. A KIM-1 assay has been developed for mouse, with a sensitivity of 2 pg/mL, in which has potential for use in urine (Sabbisetti et al., 2013).

Different procedures exist detecting and quantifying urinary NAG activity, either spectrofluorimetrically or spectrophotometrically using a wide-selection of substrates. There are available a pig N-acetyl-β-D-glucosaminidase (NAG) ELISA Kit, with a detection range of 0.156 –10 mU/mL (e.g., Cusabio ® CSB-E16198p). ELISA or a chemiluminescent microparticle immunoassay (CMIA) kits are also available for the measurement of NGAL. This biomarker is sensitive biomarker for renal damage to the proximal tubular segment of the nephron of man as well as animals. Pig NGAL ELISA Kits also exists with a detection range of 4–400 pg/mL (e.g., Biporto ® Cat: KIT 044).

Without readily available suitable methods to measure kidney biomarkers, their utility can be limited. Moreover, a wide array of kidney parameters, including novel biomarkers should be analyzed in parallel with mycotoxin metabolites. Quantification of mycotoxins in urine could be achieved by combining with specific KIM-1 tests. This would provide a point of care test for detecting nephrotoxicity caused by mycotoxins in farm animals. Urinary NAG is the predominant enzyme assay used in screening programs and is well suited for screening in low GDP countries. KIM-1 lateral-flow strip tests can be also used for screening programs (see Table 2; Wasung et al., 2015; Ráduly et al., 2021; Pócsi et al., 2022).

Rapid lateral flow tests are urgently needed for predicting AKI easily and as early as possible. For example rapid tests have been developed for human KIM-1 although the sensitivity may not be sufficient yet to predict AKI (Stojanović et al., 2015). There are some other reports in connection with KIM-1, lateral flow assays have been developed and are under trials (Vaidya et al., 2009). However, lateral flow tests need to be at least semi-quantitative, which correlates to the kidney damage caused by a toxicant (Vaidya et al., 2009).

**Table 1:** A brief summary of the suggested techniques, what biomarkers might be used for detection of mycotoxin induced nephrotoxicity.

Mycotoxin	Biomarker	Suggested technique	References
*AFB1*	*KIM-1, Cystatin C, NGAL*	*Lateral flow strip Immunoassays ELISA*	Vaidya et al. (2009) and Díaz de León-Martínez et al. (2019)
*OTA*	*KIM-1, NGAL, NAG,* β_2_-MG	*Lateral flow strip Immunoassays ELISA*	Rached et al. (2008), Vaidya et al. (2009), Yordanova et al. (2010), Hoffmann et al. (2010b), and Hassan et al. (2022)
*Fumonisins*	Sphinganine and sphingosine ratio	HPLC LC–MS (from blood spot) LC-ESI-MS, LC/MS–MS LC-FD	Merrill (1988), Seefelder et al. (2002), Schmidt et al. (2006), Silva et al. (2009), and Riley et al. (2015)

**Table 2:** Kidney biomarkers after mycotoxin exposure: a possible way to detect mycotoxin exposure.

Mycotoxin	Used Biomarker for detecting nephropathy	Sample type	References
*OTA*	*NGAL, KIM-1 (urine)*	*Mouse*	Hassan et al. (2022)
*OTA*	*KIM-1 (urine)*	*Rat*	Rached et al. (2008)
*FB_1_ + OTA*	*BUN, Cr, MDA (serum)*	*Mouse*	Li et al. (2023)
*AFB1*	*AFB1-lysine(serum) + KIM-1, Cystatin C (urine)*	*Human*	Díaz de León-Martínez et al. (2019)
*FB_1_*	Sa/So ratio (urine)	*Mouse, pig*	Kim et al. (2006) and Burel et al. (2013)

## Concluding remarks

Mycotoxins in animal feed is an increasing problem worldwide and their deleterious effects are currently underestimated. They cause potentially severe toxicological problems, affecting both man and animals. Pigs are slaughtered at the age of 6 months and develop kidney disease only very rarely. Feed testing is the routine way to control mycotoxin uptake. Kidney and liver are particularly sensitive to mycotoxin toxins and any problems in these organs compromise overall animal health status and as well meat production. Urinary kidney biomarkers are not generally analyzed in connection with mycotoxin exposure, however, this is recommended as it could help in treating as well as identifying affected animals. The mycotoxins OTA, AFB1, FB_1_ and CIT are of particular concern and their metabolites supplemented by KIM-1 would initially be a good choice for testing for mycotoxin induced nephropathy. Parallel studies with NAG and NGAL should be carried out to determine their value as screening tests. The availability of these tests would help to alleviate the increasing problem of mycotoxin contamination, but based on those tests the particular mycotoxin cannot be identified. Mycotoxin toxicity is a growing global health problem and the decrease in the amount of animal losses caused by mycotoxin exposure would have ecological as well as economic benefits. On the other hand, urinary kidney biomarkers are specific for kidney damage, but not for mycotoxin induced nephropathy alone. As a consequence, the recommended approach could be useful as a detection method when used in combination with the biomarkers of the mycotoxin metabolism excreted into the urine or blood.

## Author contributions

ZR: literature search, conceptualization, critical reading and discussion, and original draft preparation, review, and writing. LC and IP: literature search, conceptualization, funding acquisition, and critical reading, and discussion. MD: editing the manuscript, with particular focus on tubule physiology and specific biomarkers. RP: revising the English throughout the manuscript and modifying the contents of some of the section. MM, AS, and IB: literature search and critical reading and discussion. All authors contributed to the article and approved the submitted version.

## Funding

The publication was supported in Debrecen by the GINOP-2.3.2-15-2016-00062 project (IP) co-financed by the European Union and the regional Development Fund. Project no. 2018-1.2.1-NKP-2018-00002 (IP) has been implemented with the support provided from the National Research, Development, and Innovation Fund of Hungary, financed under the 2018-1.2.1-NKP funding scheme. The work was partially funded by Hungarian Academy of Sciences (ELKH-MATE 13003) and by the Hungarian National Laboratory project RRF-2.3.1-21-2022-00007 (AS). Project no. TKP2021-EGA-18 has been implemented with the support provided from the National Research, Development and Innovation Fund of Hungary, financed under the TKP2021-EGA funding scheme (ZR and LC). The publication was partially funded by The Kidney Fund (UK) core17-2022/23 (MD) and the European STEP research Programme (RP).

## Conflict of interest

The authors declare that the research was conducted in the absence of any commercial or financial relationships that could be construed as a potential conflict of interest.

## Publisher’s note

All claims expressed in this article are solely those of the authors and do not necessarily represent those of their affiliated organizations, or those of the publisher, the editors and the reviewers. Any product that may be evaluated in this article, or claim that may be made by its manufacturer, is not guaranteed or endorsed by the publisher.

## References

[ref1] Abbas H. K. Wilkinson J. R. Zablotowicz R. M. Accinelli C. Abel C. A. Bruns H. A.. (2009). Ecology of aspergillus flavus, regulation of aflatoxin production, and management strategies to reduce aflatoxin contamination of corn. Toxin Rev. 28, 142–153. doi: 10.1080/15569540903081590

[ref2] Afolabi C. G. Ojiambo P. S. Ekpo E. J. A. Menkir A. Bandyopadhyay R. (2007). Evaluation of maize inbred lines for resistance to fusarium ear rot and Fumonisin accumulation in grain in tropical Africa. Plant Dis. 91, 279–286. doi: 10.1094/PDIS-91-3-0279, PMID: 30780561

[ref3] Al Suleimani Y. M. Abdelrahman A. M. Karaca T. Manoj P. Ashique M. Nemmar A.. (2018). The effect of the dipeptidyl peptidase-4 inhibitor sitagliptin on gentamicin nephrotoxicity in mice. Biomed. Pharmacother. 97, 1102–1108. doi: 10.1016/j.biopha.2017.10.107, PMID: 29136947

[ref4] Ali N. Degen G. H. (2019). Citrinin biomarkers: a review of recent data and application to human exposure assessment. Arch. Toxicol. 93, 3057–3066. doi: 10.1007/s00204-019-02570-y, PMID: 31501918

[ref5] Ali N. Degen G. H. (2020). Biological monitoring for ochratoxin a and citrinin and their metabolites in urine samples of infants and children in Bangladesh. Mycotoxin Res 36, 409–417. doi: 10.1007/s12550-020-00407-7, PMID: 32820428

[ref6] Awuchi C. G. Ondari E. N. Ogbonna C. U. Upadhyay A. K. Baran K. Okpala C. O. R.. (2021). Mycotoxins affecting animals, foods, humans, and plants: types, occurrence, toxicities, action mechanisms, prevention, and detoxification strategies—a revisit. Foods 10:1279. doi: 10.3390/foods10061279 34205122 PMC8228748

[ref7] Battilani P. Barbano C. Marin S. Sanchis V. Kozakiewicz Z. Magan N. (2006). Mapping of aspergillus section Nigri in southern Europe and Israel based on geostatistical analysis. Int. J. Food Microbiol. 111, S72–S82. doi: 10.1016/j.ijfoodmicro.2006.03.014, PMID: 16737756

[ref8] Bennett J. W. Klich M. (2003). Mycotoxins. Clin. Microbiol. Rev. 16:497. doi: 10.1128/CMR.16.3.497-516.2003, PMID: 12857779 PMC164220

[ref9] BIOMIN. (2021) BIOMIN. BIOMIN. Available at: https://www.biomin.net/science-hub/world-mycotoxin-survey-impact-2021/ (Accessed January 15, 2023).

[ref10] Birr T. Jensen T. Preußke N. Sönnichsen F. D. De Boevre M. De Saeger S.. (2021). Occurrence of fusarium mycotoxins and their modified forms in forage maize cultivars. Toxins 13:110. doi: 10.3390/toxins13020110, PMID: 33540691 PMC7913079

[ref11] Blacutt A. A. Gold S. E. Voss K. A. Gao M. Glenn A. E. (2018). Fusarium verticillioides: advancements in understanding the toxicity, virulence, and niche adaptations of a model mycotoxigenic pathogen of maize. Phytopathology 108, 312–326. doi: 10.1094/PHYTO-06-17-0203-RVW, PMID: 28971734

[ref12] Burel C. Tanguy M. Guerre P. Boilletot E. Cariolet R. Queguiner M.. (2013). Effect of low dose of Fumonisins on pig health: immune status, intestinal microbiota and sensitivity to salmonella. Toxins 5, 841–864. doi: 10.3390/toxins5040841, PMID: 23612754 PMC3705294

[ref13] Cai Q. Tang L. Wang J. S. (2007). Validation of fumonisin biomarkers in F344 rats. Toxicol. Appl. Pharmacol. 225, 28–39. doi: 10.1016/j.taap.2007.06.027, PMID: 17904604 PMC2129219

[ref14] Chade A. R. Williams M. L. Engel J. Guise E. Harvey T. W. (2018). A translational model of chronic kidney disease in swine. Am. J. Physiol. Renal Physiol. 315, F364–F373. doi: 10.1152/ajprenal.00063.2018, PMID: 29693449 PMC6442373

[ref15] Chen Z. Zhou L. Yuan Q. Chen H. Lei H. Su J. (2021). Effect of fumonisin B1 on oxidative stress and gene expression alteration of nutrient transporters in porcine intestinal cells. J. Biochem. Mol. Toxicol. 35, 1–11. doi: 10.1002/jbt.22706, PMID: 33443779

[ref16] Corcuera L. A. Vettorazzi A. Arbillaga L. Pérez N. Gil A. G. Azqueta A.. (2015). Genotoxicity of aflatoxin B1 and Ochratoxin a after simultaneous application of the in vivo micronucleus and comet assay. Food Chem. Toxicol. 76, 116–124. doi: 10.1016/j.fct.2014.12.003, PMID: 25530104

[ref17] Díaz de León-Martínez L. Díaz-Barriga F. Barbier O. Ortíz D. L. G. Ortega-Romero M. Pérez-Vázquez F.. (2019). Evaluation of emerging biomarkers of renal damage and exposure to aflatoxin-B 1 in Mexican indigenous women: a pilot study. Environ. Sci. Pollut. Res. 26, 12205–12216. doi: 10.1007/s11356-019-04634-z, PMID: 30835068

[ref18] DSM. (2022). DSM world mycotoxin survey. The Global Threat – January to March 2022. Available at: https://www.dsm.com/anh/news/downloads/whitepapers-and-reports/q1-2022-dsm-world-mycotoxin-survey-report.html (Accessed September 10, 2022).

[ref19] Dugyala R. R. Sharma R. P. Tsunoda M. Riley R. T. (1998). Tumor necrosis factor-α as a contributor in fumonisin B1 toxicity. J. Pharmacol. Exp. Ther. 285, 317–324. 9536027

[ref20] EC (European Comission) (2006). Commission recommendation of 17 august 2006 on the presence of deoxynivalenol, zearalenone, ochratoxin a, T-2 and HT-2 and fumonisins in products intended for animal feeding. Off. J. Eur. Union 299, 7–9.

[ref21] Eckes T. Trautmann S. Djudjaj S. Beyer S. Patyna S. Schwalm S.. (2021). Consistent alteration of chain length-specific ceramides in human and mouse fibrotic kidneys. Biochim Biophys Acta Mol Cell Biol Lipids 1866:158821. doi: 10.1016/j.bbalip.2020.158821 33010454

[ref22] Ekici H. Yipel M. (2022). Total aflatoxin, aflatoxin B1, ochratoxin a and fumonisin in dry dog food: a risk assessment for dog health. Toxicon 218, 13–18. doi: 10.1016/j.toxicon.2022.08.013, PMID: 35995096

[ref23] Elling F. Hald B. Jacobsen C. Krogh P. (1975). Spontaneous toxic nephropathy in Polutry associated with Ochratoxin a. Acta Pathol. Microbiol. Scand. A Pathol. 83, 739–741. doi: 10.1111/j.1699-0463.1975.tb01406.x, PMID: 1189924

[ref24] Elling F. Nielsen J. P. Lillehøj E. B. Thomassen M. S. Størmer F. C. (1985). Ochratoxin A-induced porcine nephropathy: enzyme and ultrastructure changes after short-term exposure. Toxicon 23, 247–254. doi: 10.1016/0041-0101(85)90147-3, PMID: 4024134

[ref25] EU—COM 2014/015 (2014). Official journal. Off. J. Eur. Union 57, 1–28.

[ref26] Ezekiel C. N. Oyeyemi O. T. Oyedele O. A. Ayeni K. I. Oyeyemi I. T. Nabofa W.. (2018). Urinary aflatoxin exposure monitoring in rural and semi-urban populations in Ogun state, Nigeria. Food Addit Contamin Part A 35, 1565–1572. doi: 10.1080/19440049.2018.1475752, PMID: 29843566

[ref27] Ferenczi S. Cserháti M. Krifaton C. Szoboszlay S. Kukolya J. Szoke Z.. (2014). A new ochratoxin a biodegradation strategy using cupriavidus basilensis Or16 strain. PLoS One 9:e109817. doi: 10.1371/journal.pone.0109817, PMID: 25302950 PMC4193827

[ref28] Ferenczi S. Kuti D. Cserháti M. Krifaton C. Szoboszlay S. Kukolya J.. (2020). Effects of single and repeated Oral doses of Ochratoxin a on the lipid peroxidation and antioxidant defense Systems in Mouse Kidneys. Toxins 12:732. doi: 10.3390/toxins12110732, PMID: 33266415 PMC7700583

[ref29] Ferri F. Brera C. De Santis B. Collini G. Crespi E. Debegnach F.. (2020). Association between urinary levels of aflatoxin and consumption of food linked to maize or cow milk or dairy products. Int. J. Environ. Res. Public Health 17, 1–13. doi: 10.3390/ijerph17072510, PMID: 32268619 PMC7177871

[ref30] Flajs D. Peraica M. (2009). Toxicological properties of citrinin. Arh. Hig. Rada Toksikol. 60, 457–464. doi: 10.2478/10004-1254-60-2009-1992, PMID: 20061247

[ref31] Gambacorta S. Solfrizzo M. Visconti A. Powers S. Cossalter A. M. Pinton P.. (2013). Validation study on urinary biomarkers of exposure for aflatoxin B1, ochratoxin a, fumonisin B1, deoxynivalenol and zearalenone in piglets. World Mycotoxin J. 6, 299–308. doi: 10.3920/WMJ2013.1549

[ref32] Ganesan A. R. Mohan K. Karthick Rajan D. Pillay A. A. Palanisami T. Sathishkumar P.. (2022). Distribution, toxicity, interactive effects, and detection of ochratoxin and deoxynivalenol in food: a review. Food Chem. 378:131978. doi: 10.1016/j.foodchem.2021.131978, PMID: 35033712

[ref33] Gautier J. C. Zhou X. Yang Y. Gury T. Qu Z. Palazzi X.. (2016). Evaluation of novel biomarkers of nephrotoxicity in Cynomolgus monkeys treated with gentamicin. Toxicol. Appl. Pharmacol. 303, 1–10. doi: 10.1016/j.taap.2016.04.012, PMID: 27105553

[ref34] Grandi M. Vecchiato C. G. Biagi G. Zironi E. Tondo M. T. Pagliuca G.. (2019). Occurrence of mycotoxins in extruded commercial cat food. ACS Omega 4, 14004–14012. doi: 10.1021/acsomega.9b01702 31497718 PMC6714290

[ref35] Guerre P. Travel A. Tardieu D. (2022). Targeted analysis of sphingolipids in turkeys fed Fusariotoxins: first evidence of key changes that could help explain their relative resistance to Fumonisin toxicity. Int. J. Mol. Sci. 23:2512. doi: 10.3390/ijms23052512, PMID: 35269655 PMC8910753

[ref36] Guo Y. Balasubramanian B. Zhao Z. H. Liu W. C. (2021). Marine algal polysaccharides alleviate aflatoxin B1-induced bursa of Fabricius injury by regulating redox and apoptotic signaling pathway in broilers. Poult. Sci. 100, 844–857. doi: 10.1016/j.psj.2020.10.050, PMID: 33518138 PMC7858151

[ref37] Hall A. M. Trepiccione F. Unwin R. J. (2021). Drug toxicity in the proximal tubule: new models, methods and mechanisms. Pediatr. Nephrol. 37, 973–982. doi: 10.1007/s00467-021-05121-9, PMID: 34050397 PMC9023418

[ref38] Haschek W. M. Gumprecht L. A. Smith G. Tumbleson M. E. Constable P. D. (2001). Fumonisin toxicosis in swine: an overview of porcine pulmonary edema and current perspectives. Environ. Health Perspect. 109, 251–257. doi: 10.1289/ehp.01109s2251, PMID: 11359693 PMC1240673

[ref39] Hassan R. González D. Hobloss Z. Brackhagen L. Myllys M. Friebel A.. (2022). Inhibition of cytochrome P450 enhances the nephro- and hepatotoxicity of ochratoxin a. Arch. Toxicol. 96, 3349–3361. doi: 10.1007/s00204-022-03395-y, PMID: 36227364 PMC9584869

[ref40] Heussner A. Bingle L. Heussner A. H. Bingle L. E. H. (2015). Comparative Ochratoxin toxicity: a review of the available data. Toxins 7, 4253–4282. doi: 10.3390/toxins7104253, PMID: 26506387 PMC4626733

[ref41] Heyndrickx E. Sioen I. Huybrechts B. Callebaut A. De Henauw S. De Saeger S. (2015). Human biomonitoring of multiple mycotoxins in the Belgian population: results of the BIOMYCO study. Environ. Int. 84, 82–89. doi: 10.1016/j.envint.2015.06.011, PMID: 26233555

[ref42] Hoffmann D. Adler M. Vaidya V. S. Rached E. Mulrane L. Gallagher W. M.. (2010a). Performance of novel kidney biomarkers in preclinical toxicity studies. Toxicol. Sci. 116, 8–22. doi: 10.1093/toxsci/kfq029, PMID: 20118187 PMC2886853

[ref43] Hoffmann D. Fuchs T. C. Henzler T. Matheis K. A. Herget T. Dekant W.. (2010b). Evaluation of a urinary kidney biomarker panel in rat models of acute and subchronic nephrotoxicity. Toxicology 277, 49–58. doi: 10.1016/j.tox.2010.08.013, PMID: 20816719

[ref44] Holanda D. M. Kim S. W. (2021). Mycotoxin occurrence, toxicity, and detoxifying agents in pig production with an emphasis on Deoxynivalenol. Toxins 13.:171. doi: 10.3390/toxins13020171, PMID: 33672250 PMC7927007

[ref45] Hou L. Yuan X. Le G. Lin Z. Gan F. Li H.. (2021). Fumonisin B1 induces nephrotoxicity via autophagy mediated by mTORC1 instead of mTORC2 in human renal tubule epithelial cells. Food Chem. Toxicol. 149:112037. doi: 10.1016/j.fct.2021.112037, PMID: 33548371

[ref46] Ichimura T. Hung C. C. Yang S. A. Stevens J. L. Bonventre J. V. (2004). Kidney injury molecule-1: a tissue and urinary biomarker for nephrotoxicant-induced renal injury. Am. J. Physiol. Renal Physiol. 286, F552–F563. doi: 10.1152/ajprenal.00285.2002, PMID: 14600030

[ref47] International Agency for Research on Cancer (2012). Aflatoxins IARC monographs. Int Agency Res Cancer 100F, 225–248.

[ref48] Kamle M. Mahato D. K. Gupta A. Pandhi S. Sharma N. Sharma B.. (2022). Citrinin mycotoxin contamination in food and feed: impact on agriculture, human health, and detection and management strategies. Toxins 14:85. doi: 10.3390/toxins14020085, PMID: 35202113 PMC8874403

[ref49] Kępińska-Pacelik J. Biel W. (2021). Alimentary risk of mycotoxins for humans and animals. Toxins 13:822. doi: 10.3390/toxins13110822, PMID: 34822606 PMC8622594

[ref50] Keszthelyi S. Kadlicskó S. Pásztor G. Takács A. Szolcsányi É. Pál-Fám F.. (2022). Harvesting and phytosanitary parameters with particular regard to mycotoxin content of maize as a function of different seasonal, fertilisation and hybrid effect. Plant Soil Environ. 68, 262–271. doi: 10.17221/80/2022-PSE

[ref51] Kim D.-H. Yoo H.-S. Lee Y.-M. Kie J.-H. Jang S. Oh S. (2006). Elevation of Sphinganine 1-phosphate as a predictive biomarker for Fumonisin exposure and toxicity in mice. J. Toxic. Environ. Health A 69, 2071–2082. doi: 10.1080/15287390600746215, PMID: 17060094

[ref52] Kolawole O. Siri-Anusornsak W. Petchkongkaw A. Meneely J. Elliott C. (2022). The efficacy of additives for the mitigation of aflatoxins in animal feed: a systematic review and network meta-analysis. Toxins 14, 1–15. doi: 10.3390/toxins14100707, PMID: 36287975 PMC9607122

[ref490] Kőszegi T. Poór M. (2016). Ochratoxin A: molecular interactions, mechanisms of toxicity and prevention at the molecular level. Toxins 8:111. doi: 10.3390/toxins8040111, PMID: 27092524 PMC4848637

[ref53] Kumar P. Mahato D. K. Kamle M. Mohanta T. K. Kang S. G. (2017). Aflatoxins: a global concern for food safety, human health and their management. Front. Microbiol. 7, 1–10. doi: 10.3389/fmicb.2016.02170, PMID: 28144235 PMC5240007

[ref54] Lascano G. J. Zanton G. I. Heinrichs A. J. Weiss W. P. (2010). Technical note: a noninvasive urine collection device for female cattle: modification of the urine cup collection method. J. Dairy Sci. 93, 2691–2694. doi: 10.3168/jds.2009-3027, PMID: 20494178

[ref55] Lee H. J. Pyo M. C. Shin H. S. Ryu D. Lee K. W. (2018). Renal toxicity through AhR, PXR, and Nrf2 signaling pathway activation of ochratoxin A-induced oxidative stress in kidney cells. Food Chem. Toxicol. 122, 59–68. doi: 10.1016/j.fct.2018.10.004, PMID: 30291945

[ref56] Leslie J. F. Moretti A. Mesterh Á. Ameye M. Audenaert K. Singh P. K.. (2021). Key global actions for mycotoxin management in wheat and other small grains. Toxins 13:725. doi: 10.3390/toxins13100725 34679018 PMC8541216

[ref57] Leszkowicz A. Molinie A. Tozlovanu M. Manderville R. (2008). “Combined toxic effects of ochratoxin a and Citrinin, *In Vivo and In Vitro*,” in Food Contaminants, Mycotoxins and Food Allergens, Vol. 1001, eds. P. Darsa, M. W. Siantar, P. E. Trucksess, Scott, and E. M. Herman (Washington, DC: ACS Publication), 56–79.

[ref58] Li H. He W. Yue D. Wang M. Yuan X. Huang K. (2023). Low doses of fumonisin B1 exacerbate ochratoxin A-induced renal injury in mice and the protective roles of heat shock protein 70. Chem. Biol. Interact. 369:110240. doi: 10.1016/j.cbi.2022.110240, PMID: 36397609

[ref59] Li H. Xing L. Zhang M. Wang J. Zheng N. (2018). The toxic effects of aflatoxin B1 and aflatoxin M1 on kidney through regulating L-proline and downstream apoptosis. Biomed. Res. Int. 2018, 1–11. doi: 10.1155/2018/9074861, PMID: 30159329 PMC6109566

[ref60] Lipcsey M. Carlsson M. Larsson A. Algotsson L. Eriksson M. Lukinius A.. (2009). Effect of a single dose of tobramycin on systemic inflammatory response-induced acute kidney injury in a 6-hour porcine model *. Crit. Care Med. 37, 2782–2790. doi: 10.1097/00003246-200910000-00016 19707126

[ref61] Liu W. C. Pushparaj K. Meyyazhagan A. Arumugam V. A. Pappuswamy M. Bhotla H. K.. (2022a). Ochratoxin a as an alarming health threat for livestock and human: a review on molecular interactions, mechanism of toxicity, detection, detoxification, and dietary prophylaxis. Toxicon 213, 59–75. doi: 10.1016/j.toxicon.2022.04.012, PMID: 35452686

[ref62] Liu W. C. Yang Y. Y. Pushparaj K. Balasubramanian B. (2022b). Evaluation of hepatic detoxification effects of Enteromorpha prolifera polysaccharides against aflatoxin B1 in broiler chickens. Antioxidants 11, 1–13. doi: 10.3390/antiox11091757, PMID: 36139831 PMC9495745

[ref63] Loiseau N. Polizzi A. Dupuy A. Therville N. Rakotonirainy M. Loy J.. (2015). New insights into the organ-specific adverse effects of fumonisin B1: comparison between lung and liver. Arch. Toxicol. 89, 1619–1629. doi: 10.1007/s00204-014-1323-6, PMID: 25155190

[ref64] Malir F. Louda M. Ostry V. Toman J. Ali N. Grosse Y.. (2019). Analyses of biomarkers of exposure to nephrotoxic mycotoxins in a cohort of patients with renal tumours. Mycotoxin Res 35, 391–403. doi: 10.1007/s12550-019-00365-9, PMID: 31254204

[ref65] Manafi M. Mohan K. Mohmand N. A. (2011). Effect of ochratoxin a on coccidiosis-challenged broiler chicks. World Mycotoxin J. 4, 177–181. doi: 10.3920/WMJ2010.1234

[ref66] Marczak L. Idkowiak J. Tracz J. Stobiecki M. Perek B. Kostka-Jeziorny K.. (2021). Mass spectrometry-based lipidomics reveals differential changes in the accumulated lipid classes in chronic kidney disease. Meta 11, 1–22. doi: 10.3390/metabo11050275, PMID: 33925471 PMC8146808

[ref67] Marin-Kuan M. Nestler S. Verguet C. Bezençon C. Piguet D. Mansourian R.. (2006). A toxicogenomics approach to identify new plausible epigenetic mechanisms of ochratoxin a carcinogenicity in rat. Toxicol. Sci. 89, 120–134. doi: 10.1093/toxsci/kfj017, PMID: 16251485

[ref68] Martínez-de-Anda A. Valdivia A. G. Jaramillo-Juárez F. Reyes J. L. Ortiz R. Quezada T.. (2010). Effects of aflatoxin chronic intoxication in renal function of laying hens. Poult. Sci. 89, 1622–1628. doi: 10.3382/ps.2010-00763, PMID: 20634516

[ref69] Meerpoel C. Vidal A. Tangni E. K. Huybrechts B. Couck L. De Rycke R.. (2020). A study of carry-over and histopathological effects after chronic dietary intake of Citrinin in pigs, broiler chickens and laying hens. Toxins 12, 1–19. doi: 10.3390/toxins12110719, PMID: 33207646 PMC7697729

[ref70] Meredith F. I. Riley R. T. Bacon C. W. Williams D. E. Carlson D. B. (1998). Extraction, quantification, and biological availability of fumonisin B1 incorporated into the Oregon test diet and fed to rainbow trout. J. Food Prot. 61, 1034–1038. doi: 10.4315/0362-028X-61.8.1034, PMID: 9713767

[ref71] Merrill A. H. (1988). Quantitaion of free sphingosine in liver by HPLC. Anal. Biochem. 171, 373–381. doi: 10.1016/0003-2697(88)90500-3, PMID: 3407935

[ref72] Merrill A. H. Van Echten G. Wang E. Sandhoff K. (1993). Fumonisin B1 inhibits sphingosine (sphinganine) N-acyltransferase and de novo sphingolipid biosynthesis in cultured neurons in situ. J. Biol. Chem. 268, 27299–27306. doi: 10.1016/s0021-9258(19)74249-5, PMID: 8262970

[ref73] Mohd Redzwan S. Rosita J. Mohd Sokhini A. M. Nurul ‘Aqilah A. R. Wang J. S. Kang M. S.. (2014). Detection of serum AFB1-lysine adduct in Malaysia and its association with liver and kidney functions. Int. J. Hyg. Environ. Health 217, 443–451. doi: 10.1016/j.ijheh.2013.08.007, PMID: 24095591

[ref75] Musiał K. (2021). Current concepts of pediatric acute kidney injury—are we ready to translate them into everyday practice? J. Clin. Med. 10:3113. doi: 10.3390/jcm10143113, PMID: 34300278 PMC8305016

[ref76] Nasir U. Naeem I. Asif M. Ismail A. Gong Y. Y. Routledge M. N.. (2021). Assessment of aflatoxins exposure through urinary biomarker approach and the evaluation of the impacts of aflatoxins exposure on the selected health parameters of the children of Multan city of Pakistan. Food Control 123:107863. doi: 10.1016/j.foodcont.2021.107863

[ref77] Naudé P. J. W. Mommersteeg P. M. C. Gouweleeuw L. Eisel U. L. M. Denollet J. Westerhuis L. W. J. J. M.. (2015). NGAL and other markers of inflammation as competitive or complementary markers for depressive symptom dimensions in heart failure. World J. Biol. Psychiatry 16, 536–541. doi: 10.3109/15622975.2015.1062550, PMID: 26212793

[ref78] Nesbitt B. F. O’Kelly J. Sargeant K. Sheridan A. N. N. (1962). Aspergillus Flavus and Turkey X disease: toxic metabolites of aspergillus flavus. Nature 195, 1062–1063. doi: 10.1038/1951062a0, PMID: 14479064

[ref79] Nigam S. K. Wu W. Bush K. T. Hoenig M. P. Blantz R. C. Bhatnagar V. (2015). Handling of drugs, metabolites, and uremic toxins by kidney proximal tubule drug transporters. Clin. J. Am. Soc. Nephrol. 10, 2039–2049. doi: 10.2215/CJN.02440314, PMID: 26490509 PMC4633783

[ref80] Norred W. P. Plattner R. D. Chamberlain W. J. (1993). Distribution and excretion of [14C] fumonisin B1 in male Sprague-dawley rats. Nat. Toxins 1, 341–346. doi: 10.1002/nt.2620010604, PMID: 8167955

[ref81] Ostry V. Malir F. Ruprich J. (2013). Producers and important dietary sources of ochratoxin a and citrinin. Toxins 5, 1574–1586. doi: 10.3390/toxins5091574, PMID: 24048364 PMC3798874

[ref82] Ostry V. Malir F. Toman J. Grosse Y. (2017). Mycotoxins as human carcinogens—the IARC monographs classification. Mycotoxin Res 33, 65–73. doi: 10.1007/s12550-016-0265-7, PMID: 27888487

[ref83] Park S. Koo J. Kim B. Pushparaj K. Malaisamy A. Liu W. C.. (2022). Evaluation of the safety and Ochratoxin a degradation capacity of Pediococcus pentosaceus as a dietary probiotic with molecular docking approach and pharmacokinetic toxicity assessment. Int. J. Mol. Sci. 23:9062. doi: 10.3390/ijms23169062, PMID: 36012326 PMC9409003

[ref84] Patriarca A. Fernández P. V. (2017). Prevalence of mycotoxins in foods and decontamination. Curr. Opin. Food Sci. 14, 50–60. doi: 10.1016/j.cofs.2017.01.011

[ref85] Perrone G. Haidukowski M. Stea G. Epifani F. Bandyopadhyay R. Leslie J. F.. (2014). Population structure and aflatoxin production by aspergillus sect. Flavi from maize in Nigeria and Ghana. Food Microbiol. 41, 52–59. doi: 10.1016/j.fm.2013.12.005, PMID: 24750813

[ref86] Pickova D. Ostry V. Toman J. Malir F. (2021). Aflatoxins: history, significant milestones, recent data on their toxicity and ways to mitigation. Toxins 13, 1–23. doi: 10.3390/toxins13060399, PMID: 34205163 PMC8227755

[ref87] Pócsi I. Dockrell M. E. Price R. G. (2022). Nephrotoxic biomarkers with specific indications for metallic pollutants: implications for environmental health. Biomark. Insights 17:11772719221111882. doi: 10.1177/11772719221111882, PMID: 35859925 PMC9290154

[ref88] Prietto L. Moraes P. S. Kraus R. B. Meneghetti V. Fagundes C. A. A. Furlong E. B. (2015). Post-harvest operations and aflatoxin levels in rice (Oryza sativa). Crop Prot. 78, 172–177. doi: 10.1016/j.cropro.2015.09.011

[ref89] Rached E. Hoffmann D. Blumbach K. Weber K. Dekant W. Mally A. (2008). Evaluation of putative biomarkers of nephrotoxicity after exposure to ochratoxin a in vivo and in vitro. Toxicol. Sci. 103, 371–381. doi: 10.1093/toxsci/kfn040, PMID: 18308701

[ref90] Ráduly Z. Price R. G. Dockrell M. E. C. Csernoch L. Pócsi I. (2021). Urinary biomarkers of mycotoxin induced nephrotoxicity–current status and expected future trends. Toxins 13:848. doi: 10.3390/toxins13120848, PMID: 34941686 PMC8708607

[ref91] Rheeder J. P. Marasas W. F. O. Vismer H. F. (2002). Production of fumonisin analogs by fusarium species. Appl. Environ. Microbiol. 68, 2101–2105. doi: 10.1128/AEM.68.5.2101-2105.2002, PMID: 11976077 PMC127586

[ref92] Riley R. T. Merrill A. H. (2019). Ceramide synthase inhibition by fumonisins: a perfect storm of perturbed sphingolipid metabolism, signaling, and disease. J. Lipid Res. 60, 1183–1189. doi: 10.1194/jlr.S093815, PMID: 31048407 PMC6602133

[ref93] Riley R. T. Showker J. L. Lee C. M. Zipperer C. E. Mitchell T. R. Voss K. A.. (2015). A blood spot method for detecting fumonisin-induced changes in putative sphingolipid biomarkers in LM/Bc mice and humans. Food Addit Contaminants Part A 32, 934–949. doi: 10.1080/19440049.2015.1027746, PMID: 25833119

[ref94] Riley R. T. Wang E. Merrill A. H. Jr. (1994). Liquid chromatographic determination of Sphmgamne and sphingosine: use of the free Sphinganine-to-sphingosine ratio as a biomarker for consumption of Fumonisins. J. AOAC Int. 77, 533–540. doi: 10.1093/jaoac/77.2.533

[ref95] Ringot D. Chango A. Schneider Y. J. Larondelle Y. (2006). Toxicokinetics and toxicodynamics of ochratoxin a, an update. Chem. Biol. Interact. 159, 18–46. doi: 10.1016/j.cbi.2005.10.106, PMID: 16293235

[ref96] Rushing B. R. Selim M. I. (2019). Aflatoxin B1: a review on metabolism, toxicity, occurrence in food, occupational exposure, and detoxification methods. Food Chem. Toxicol. 124, 81–100. doi: 10.1016/j.fct.2018.11.047, PMID: 30468841

[ref97] Sabbisetti V. S. Ito K. Wang C. Yang L. Mefferd S. C. Bonventre J. V. (2013). Novel assays for detection of urinary KIM-1 in mouse models of kidney injury. Toxicol. Sci. 131, 13–25. doi: 10.1093/toxsci/kfs268, PMID: 23019274 PMC3621351

[ref98] Sakuda S. Prabowo D. F. Takagi K. Shiomi K. Mori M. Omura S.. (2014). Inhibitory effects of respiration inhibitors on aflatoxin production. Toxins 6, 1193–1200. doi: 10.3390/toxins6041193, PMID: 24674936 PMC4014728

[ref99] Schertz H. Dänicke S. Frahm J. Schatzmayr D. Dohnal I. Bichl G.. (2018). Biomarker evaluation and toxic effects of an acute oral and systemic fumonisin exposure of pigs with a special focus on dietary fumonisin esterase supplementation. Toxins 10:296. doi: 10.3390/toxins10070296, PMID: 30018261 PMC6071024

[ref100] Schiffl H. Lang S. M. (2012). Update on biomarkers of acute kidney injury. Mol. Diagn. Ther. 16, 199–207. doi: 10.1007/bf03262209 22650449

[ref101] Schmidt H. Schmidt R. Geisslinger G. (2006). LC-MS/MS-analysis of sphingosine-1-phosphate and related compounds in plasma samples. Prostaglandins Other Lipid Mediat. 81, 162–170. doi: 10.1016/j.prostaglandins.2006.09.003, PMID: 17085324

[ref102] Schulz M. C. Schumann L. Rottkord U. Humpf H. U. Gekle M. Schwerdt G. (2018). Synergistic action of the nephrotoxic mycotoxins ochratoxin a and citrinin at nanomolar concentrations in human proximal tubule-derived cells. Toxicol. Lett. 291, 149–157. doi: 10.1016/j.toxlet.2018.04.014, PMID: 29673704

[ref103] Schumacher J. Moll H. D. (2011). Manual of equine diagnostic procedures, collecting urine. Teton New Media, Jackson. International Veterinary Information Service (IVIS.ORG).

[ref104] Seefelder W. Schwerdt G. Freudinger R. Gekle M. Humpf H. U. (2002). Liquid chromatography/electrospray ionisation-mass spectrometry method for the quantification of sphingosine and sphinganine in cell cultures exposed to fumonisins. J. Chromatogr. B Analyt. Technol. Biomed. Life Sci. 780, 137–144. doi: 10.1016/S1570-0232(02)00440-3, PMID: 12383489

[ref105] Seeling K. Dänicke S. Lebzien P. Valenta H. Ueberschär K. H. Flachowsky G. (2005). On the effects of *Fusarium*-contaminated wheat and the feed intake level on ruminal fermentation and toxin-turnover of cows. Mycotox. Res. 21, 132–135. doi: 10.1007/BF02954437 23605275

[ref106] Sheira G. Noreldin N. Tamer A. Saad M. (2015). Urinary biomarker N-acetyl-β-D-glucosaminidase can predict severity of renal damage in diabetic nephropathy. J. Diabetes Metab. Disord. 14, 4–5. doi: 10.1186/s40200-015-0133-6, PMID: 25717442 PMC4340101

[ref107] Shephard G. S. Thiel P. G. Sydenham E. W. Vleggaar R. Alberts J. F. (1994). Determination of the mycotoxin fumonisin B1 and identification of its partially hydrolysed metabolites in the faeces of non-human primates. Food Chem. Toxicol. 32, 23–29. doi: 10.1016/0278-6915(84)90032-2, PMID: 8132161

[ref108] Silva L. J. G. Lino C. M. Pena A. (2009). Sphinganine-sphingosine ratio in urine from two Portuguese populations as biomarker to fumonisins exposure. Toxicon 54, 390–398. doi: 10.1016/j.toxicon.2009.05.011, PMID: 19477192

[ref109] Solcan C. Timofte D. Floristean V. Carter S. D. Solcan G. (2013). Ultrastructural lesions and immunohistochemical analysis of Bcl-2 protein expression in the kidney of chickens with experimental ochratoxicosis. Acta Vet. Hung. 61, 344–353. doi: 10.1556/avet.2013.021, PMID: 23921346

[ref110] Song J. Yu J. Prayogo G. W. Cao W. Wu Y. Jia Z.. (2019). Understanding kidney injury molecule 1: a novel immune factor in kidney pathophysiology. Am. J. Transl. Res. 11, 1219–1229. 30972157 PMC6456506

[ref111] Stojanović V. D. Barišić N. A. Vučković N. M. Doronjski A. D. Peco Antić A. E. (2015). Urinary kidney injury molecule-1 rapid test predicts acute kidney injury in extremely low-birth-weight neonates. Pediatr. Res. 78, 430–435. doi: 10.1038/pr.2015.125, PMID: 26107391

[ref112] Tkaczyk A. Jedziniak P. Zielonka Ł. Dąbrowski M. Ochodzki P. Rudawska A. (2021). Biomarkers of deoxynivalenol, citrinin, ochratoxin a and zearalenone in pigs after exposure to naturally contaminated feed close to guidance values. Toxins 13:750. doi: 10.3390/toxins13110750, PMID: 34822534 PMC8625168

[ref113] Togashi Y. Sakaguchi Y. Miyamoto M. Miyamoto Y. (2012). Urinary cystatin C as a biomarker for acute kidney injury and its immunohistochemical localization in kidney in the CDDP-treated rats. Exp. Toxicol. Pathol. 64, 797–805. doi: 10.1016/j.etp.2011.01.018, PMID: 21377848

[ref114] Tonomura Y. Tsuchiya N. Torii M. Uehara T. (2010). Evaluation of the usefulness of urinary biomarkers for nephrotoxicity in rats. Toxicology 273, 53–59. doi: 10.1016/j.tox.2010.04.015, PMID: 20438795

[ref115] Torres A. M. Barros G. G. Palacios S. A. Chulze S. N. Battilani P. (2014). Review on pre- and post-harvest management of peanuts to minimize aflatoxin contamination. Food Res. Int. 62, 11–19. doi: 10.1016/j.foodres.2014.02.023

[ref116] Tran S. T. Tardieu D. Auvergne A. Bailly J. D. Babilé R. Durand S.. (2006). Serum sphinganine and the sphinganine to sphingosine ratio as a biomarker of dietary fumonisins during chronic exposure in ducks. Chem. Biol. Interact. 160, 41–50. doi: 10.1016/j.cbi.2005.07.009, PMID: 16413517

[ref117] Turner P. C. Snyder J. A. (2021). Development and limitations of exposure biomarkers to dietary contaminants mycotoxins. Toxins 13:314. doi: 10.3390/toxins13050314, PMID: 33924868 PMC8147022

[ref118] Udomkun P. Wiredu A. N. Nagle M. Müller J. Vanlauwe B. Bandyopadhyay R. (2017). Innovative technologies to manage aflatoxins in foods and feeds and the profitability of application – a review. Food Control 76, 127–138. doi: 10.1016/J.FOODCONT.2017.01.008, PMID: 28701823 PMC5484778

[ref119] Vaidya V. S. Ford G. M. Waikar S. S. Wang Y. Clement M. B. Ramirez V.. (2009). A rapid urine test for early detection of kidney injury. Kidney Int. 76, 108–114. doi: 10.1038/ki.2009.96, PMID: 19387469 PMC2737345

[ref120] Vaidya V. S. Ozer J. S. Dieterle F. Collings F. B. Ramirez V. Troth S.. (2010). Kidney injury molecule-1 outperforms traditional biomarkers of kidney injury in preclinical biomarker qualification studies. Nat. Biotechnol. 28, 478–485. doi: 10.1038/nbt.1623, PMID: 20458318 PMC2885849

[ref121] Vanmassenhove J. Vanholder R. Nagler E. Van Biesen W. (2013). Urinary and serum biomarkers for the diagnosis of acute kidney injury: an in-depth review of the literature. Nephrol. Dial. Transplant. 28, 254–273. doi: 10.1093/ndt/gfs380, PMID: 23115326

[ref122] Vettorazzi A. van Delft J. López de Cerain A. (2013). A review on ochratoxin a transcriptomic studies. Food Chem. Toxicol. 59, 766–783. doi: 10.1016/j.fct.2013.05.043, PMID: 23747715

[ref123] Vlachou M. Pexara A. Solomakos N. Govaris A. (2022). Ochratoxin a in slaughtered pigs and pork products. Toxins 14:67. doi: 10.3390/toxins14020067, PMID: 35202095 PMC8876995

[ref124] Vlasakova K. Troth S. P. Sistare F. D. Glaab W. E. (2020). Evaluation of 10 urinary biomarkers for renal safety with 5 Nephrotoxicants in nonhuman primates. Toxicol. Pathol. 48, 633–648. doi: 10.1177/0192623320932159, PMID: 32633702

[ref125] Wang X. Wu Q. Wan D. Liu Q. Chen D. Liu Z.. (2015). Fumonisins: oxidative stress-mediated toxicity and metabolism in vivo and in vitro. Arch. Toxicol. 90, 81–101. doi: 10.1007/s00204-015-1604-8, PMID: 26419546

[ref126] Wangia R. N. Githanga D. P. Xue K. S. Tang L. Anzala O. A. Wang J. S. (2019). Validation of urinary sphingolipid metabolites as biomarker of effect for fumonisins exposure in Kenyan children. Biomarkers 24, 379–388. doi: 10.1080/1354750X.2019.1587510, PMID: 30821509

[ref127] Wasung M. E. Chawla L. S. Madero M. (2015). Biomarkers of renal function, which and when? Clin. Chim. Acta 438, 350–357. doi: 10.1016/j.cca.2014.08.039 25195004

[ref128] Watson S. Moore S. E. Darboe M. K. Chen G. Tu Y. K. Huang Y. T.. (2018). Impaired growth in rural Gambian infants exposed to aflatoxin: a prospective cohort study. BMC Public Health 18, 1–9. doi: 10.1186/s12889-018-6164-4, PMID: 30413157 PMC6234772

[ref129] Weidemann D. K. Weaver V. M. Fadrowski J. J. (2016). Toxic environmental exposures and kidney health in children. Pediatr. Nephrol. 31, 2043–2054. doi: 10.1007/s00467-015-3222-3, PMID: 26458883 PMC4829489

[ref130] Yang D. Ye Y. Sun J. Wang J.-S. Huang C. Sun X. (2022). Occurrence, transformation, and toxicity of fumonisins and their covert products during food processing. Crit. Rev. Food Sci. Nutr. 1–14, 1–14. doi: 10.1080/10408398.2022.2134290, PMID: 36239314

[ref131] Yarru L. P. Settivari R. S. Antoniou E. Ledoux D. R. Rottinghaus G. E. (2009). Toxicological and gene expression analysis of the impact of aflatoxin B1 on hepatic function of male broiler chicks. Poult. Sci. 88, 360–371. doi: 10.3382/ps.2008-00258, PMID: 19151351

[ref132] Yordanova P. Wilfried K. Tsolova S. Dimitrov P. (2010). Ochratoxin a and β2-microglobulin in BEN patients and controls. Toxins 2, 780–792. doi: 10.3390/toxins2040780 22069610 PMC3153209

[ref133] Zachariasova M. Dzuman Z. Veprikova Z. Hajkova K. Jiru M. Vaclavikova M.. (2014). Occurrence of multiple mycotoxins in European feedingstuffs, assessment of dietary intake by farm animals. Anim. Feed Sci. Technol. 193, 124–140. doi: 10.1016/j.anifeedsci.2014.02.007

